# Physicochemical, Pharmacokinetic, and Toxicity Evaluation of Soluplus^®^ Polymeric Micelles Encapsulating Fenbendazole

**DOI:** 10.3390/pharmaceutics12101000

**Published:** 2020-10-21

**Authors:** Ik Sup Jin, Min Jeong Jo, Chun-Woong Park, Youn Bok Chung, Jin-Seok Kim, Dae Hwan Shin

**Affiliations:** 1 College of Pharmacy, Chungbuk National University, Cheongju 28160, Korea; iksup0418@gmail.com (I.S.J.); cmj0310@naver.com (M.J.J.); cwpark@chungbuk.ac.kr (C.-W.P.); chungyb@chungbuk.ac.kr (Y.B.C.); 2 Drug Information Research Institute (DIRI), College of Pharmacy, Sookmyung Women’s University, Cheongpa-ro 47-gil 100, Yongsan-gu, Seoul 04310, Korea; jsk9574@sookmyung.ac.kr

**Keywords:** fenbendazole, micelle solubilization, pharmacokinetics, Soluplus^®^ polymeric micelles, toxicity test

## Abstract

Fenbendazole (FEN), a broad-spectrum benzimidazole anthelmintic, suppresses cancer cell growth through various mechanisms but has low solubility and achieves low blood concentrations, which leads to low bioavailability. Solubilizing agents are required to prepare poorly soluble drugs for injections; however, these are toxic. To overcome this problem, we designed and fabricated low-toxicity Soluplus^®^ polymeric micelles encapsulating FEN and conducted toxicity assays in vitro and in vivo. FEN-loaded Soluplus^®^ micelles had an average particle size of 68.3 ± 0.6 nm, a zeta potential of −2.3 ± 0.2 mV, a drug loading of 0.8 ± 0.03%, and an encapsulation efficiency of 85.3 ± 2.9%. MTT and clonogenic assays were performed on A549 cells treated with free FEN and FEN-loaded Soluplus^®^ micelles. The in vitro drug release profile showed that the micelles released FEN more gradually than the solution. Pharmacokinetic studies revealed lower total clearance and volume of distribution and higher area under the curve and plasma concentration at time zero of FEN-loaded Soluplus^®^ micelles than of the FEN solution. The in vivo toxicity assay revealed that FEN-loaded Soluplus^®^ micelle induced no severe toxicity. Therefore, we propose that preclinical and clinical safety and efficacy trials on FEN-loaded Soluplus^®^ micelles would be worthwhile.

## 1. Introduction

Certain cancers have high mortality rates in humans. Therefore, the development of safe anticancer drugs with few side effects is an important research objective in pharmaceutical science. Fenbendazole (FEN) is a broad-spectrum benzimidazole anthelmintic. It has been widely used in veterinary medicine and induces no significant side effects. The European Medicines Agency has not established a no observable adverse effects level (NOAEL) for single-dose FEN administration but recommended a NOAEL of 4 mg kg^−1^ BW d^−1^ for repeated FEN administration. Moreover, no significant side effects were observed in humans in response to the administration of the major FEN metabolite oxfendazole even at 60 mg kg^−1^ for 14 d [1]. Benzimidazole (BZD) anthelmintic agents have been studied in recent years for their anticancer effects [2,3]. Several papers have reported their modes of action and positive effects [4,5]. FEN suppresses cancer cell growth through various mechanisms. It inhibits proteasomal activity and induces endoplasmic reticulum stress and reactive oxygen species-dependent apoptosis [6]. Second, it has anti-tubulin efficacy [5]. Other BZDs arrest mitosis and promote apoptosis. Mebendazole displays anti-tubulin and antitumor efficacy in vivo [7]. However, FEN has low solubility and achieves only low blood concentrations [8,9]. The limited solubility makes it difficult to develop parenteral and even topical preparations. For these reasons, it has a low area under the curve (AUC) and poor bioavailability [10]. AUC is an intuitive indicator of how much body has been exposed to drugs and is also strongly related to drug efficacy and number of drug administration. Therefore, it is important to increase the solubility of FEN, which can lead to improved bioavailability [11,12]. Hence, injectable forms of FEN are preferable as they can bypass the various obstacles of the digestive tract. However, solubilizing agent is needed to prepare poorly soluble drugs for injections. Ethanol (EtOH) and Cremophor EL^®^ increase paclitaxel solubility. However, Cremophor EL may induce peripheral neurotoxicity, neutropenia, and hypersensitivity reactions [13,14,15]. Another solubilizing agent, polysorbate 80 (Tween 80^®^), which exerts acute toxicity in an in vivo zebrafish model, is used in Taxotere^®^, an anticancer drug containing docetaxel [16]. Finally, dimethylacetamide (DMA), used in anticancer drug Vunom^®^ containing teniposide, results in hepatic fatty infiltration, liver hypertrophy, and focal hepatic necrosis in animals, and acute hepatitis has been reported in humans [17,18,19]. There is a formulation technology using nanoparticles to replace such a solubilizing agent. Examples of nanoparticles include liposomes, microemulsions, and micelles [20,21,22]. We designed and tested various micelles composed of amphiphilic diblock copolymers to enhance FEN solubilization while mitigating the toxicity of the different excipients used for this purpose [23]. The enhanced permeability and retention (EPR) effect of nanomicelles increases their accumulation in cancer cells. The EPR effect is a phenomenon manifested by the peculiar pathophysiological characteristics of solid tumors. Nanoparticles are trapped in the solid tumors and stay longer for reasons such as hyper vasculature and impaired lymphatic drainage/recovery system [24]. In addition, polymeric micelles are biodegradable, biocompatible, have low toxicity, and may have nearly solid inner cores that can serve as drug carriers [25]. We found four suitable candidate polymers to fabricate the micelles. Each polymer has both specific and general nanoparticle advantages. The mPEG-*b*-PLA micelle can hold and carry multiple drugs [26]. This polymer can also remain in circulation for a long time and has low toxicity [27]. In cancer treatment, the occurrence of drug resistance owing to P-glycoprotein (P-gp) overexpression is a serious problem [28]. However, Soluplus^®^ might be able to inhibit P-gp [29]. Paclitaxel encapsulated with Pluronic^®^ F127 overcomes multidrug resistance (MDR) and has favorable pharmacokinetics. Intracellular ATP depletion and lowered mitochondrial potential are assumed to be involved in the modulation of MDR [30].

**Figure 1:**
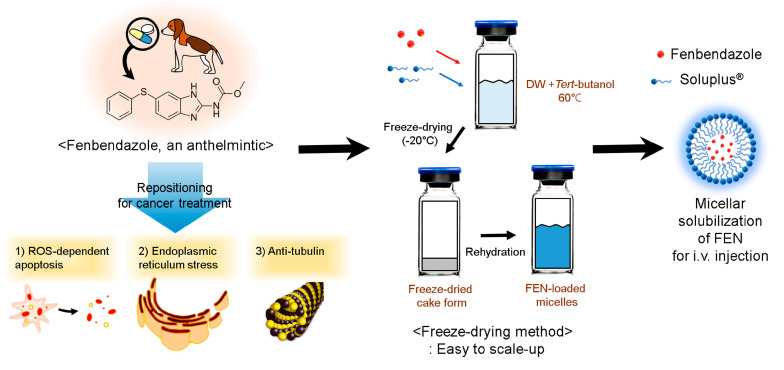
Schematic illustration of fenbendazole (FEN)-loaded Soluplus^®^ micelle preparation for i.v. injection.

In this study, we selected the most suitable polymer and fabricated micelles using the freeze-drying method. This is advantageous for scaled-up production, easy storage, and cake-state distribution. Then, we evaluated the in vitro cytotoxicity, in vivo toxicity, in vitro release, and pharmacokinetic profile. Our findings will help to develop a potent and innovative injectable micellar formulation facilitating the clinical study and application of FEN for anticancer purposes (Figure 1).

## 2. Materials and Methods

### 2.1. Materials and Reagents

Soluplus^®^ (Polyvinyl caprolactam-polyvinyl acetate-polyethylene glycol graft copolymer (PCL-PVAc-PEG)), was kindly donated by BASF (Ludwigshafen, Rhineland-Palatinate, Germany). Methoxy poly(ethylene glycol)-*b*-poly(d,l-lactide) (mPEG [4000]-*b*-PLA [2200]) was purchased from Advanced Polymer Materials, Inc. (Montreal, QC, Canada). Poly(ethylene oxide-*b*-ε-caprolactone) (PEO[5000]-*b*-PCL[10000]) was purchased from Polymer Source, Inc. (Montreal, QC, Canada). Pluronic^®^ F127 was purchased from Sigma-Aldrich Corp. (St. Louis, MO, USA). Dulbecco’s phosphate-buffered saline (DPBS), and trypsin were purchased from Corning Inc. (Corning, NY, USA). EtOH and acetonitrile (ACN) were obtained from Thermo Fisher Scientific (Waltham, MA, USA). Distilled water (DW) was purchased from Tedia (Fairfield, OH, USA). FEN, Thiazolyl blue tetrazolium bromide (MTT), Dimethyl sulfoxide (DMSO), Polysorbate 80 (Tween 80^®^), DMA, and Cremophor EL^®^ were purchased from Sigma-Aldrich Corp. (St. Louis, MO, USA). All other reagents were of at least analytical or HPCL grade.

### 2.2. Methods

#### 2.2.1. Preparation of FEN-Loaded Polymeric Micelles

FEN-loaded polymeric micelles were prepared from four polymers by the freeze-drying method [31]. Briefly, 1 mg of FEN and 100 mg of polymers were dissolved in 1 mL of tert-butanol and stirred for 1 min in prewarmed water at 60 °C. Then 1 mL of DW was added and the mixture was vortexed for 1 min. The mixture was rapidly frozen at −70 °C for 1 h, placed in a freeze-dryer (Advantage Pro; SP Scientific, Warminster, PA, USA), and lyophilized for 24 h. Then, 1 mL of DW at 60 °C was added to hydrate the mixture. The solution was then centrifuged at 16,600× *g* at 4 °C (Hanil Science Inc., Gimpo, Korea) for 5 min to remove drug or polymer precipitates. A non-pyrogenic sterile syringe filter with 0.2 µm pore size (Corning, NY, USA) was used to further remove remaining debris and to make sterile condition [32,33].

#### 2.2.2. High-Performance Liquid Chromatography (HPLC) Analysis

A HPLC system was used for the analysis of concentrations of FEN. All samples were obtained from the in vitro and in vivo assays through this study. The HPLC system consisted of Waters 2695 separation module and a Waters 2996 photodiode array detector (Waters, Milford, MA, USA). A Fortis C18 chromatography column (5 µm, 4.6 × 250 mm) (Fortis Technologies Ltd., Cheshire, UK) was run in a 30 °C environment. FEN and a genistein internal standard (IS) were eluted in the isocratic mode. The mobile phase consisted of water/ACN (30:70, *v*/*v*), and it was replaced and degassed for each run. The sample injection volume was 10 µL, and the mobile phase flow rate was 1.0 mL min^−1^. The IS and FEN retention times were 3.4 and 5.4 min, respectively (Supplementary Materials Figure S1). The predetermined calibration curve was used to calculate the concentrations. The concentration was calculated by substituting the peak area of each sample into the calibration curve.

#### 2.2.3. Physicochemical Micelle Characterization

A dynamic light scattering (DLS) device (Litesizer 500, Anton Paar, Graz, Austria) measured the FEN-loaded micelles zeta-potentials and particle sizes. The angle of measurement was automatically selected and used between side scatter (90°) and back scatter (175°). The FEN-loaded micelle encapsulation efficiency (EE, %) and drug loading (DL, %) were obtained by HPLC and calculated as follows:

DL% = weight of drug in micelles/weight of feeding polymer and drug × 100(1)

EE% = weight of drug in micelles/weight of feeding drug × 100(2)

The results of each sample analysis are expressed as the mean ± standard deviation of three separate experiments.

#### 2.2.4. Transmission Electron Microscopy Study

Images of micelles were obtained using a transmission electron microscope (TEM) (JEM-2100 Plus, JEOL, Tokyo, Japan). Diluted micelle suspensions were dropped onto 200-mesh formvar-coated copper grids. After loaded, it was dried at 60 °C in a dry oven for 12 h. Finally, FEN-loaded Soluplus^®^ micelles were measured using a TEM operated at 200 kV.

#### 2.2.5. In Vitro Drug Release Assay

Dialysis with a phosphate-buffered saline (PBS, pH 7.4) medium was used to investigate the in vitro drug release patterns. In brief, FEN-loaded micelles and FEN solution were inserted into a dialysis membrane bag (molecular weight cut-off = 20 kDa), which was then tied and immersed in 2.0 L of medium maintained at 37 °C and stirred constantly with a magnetic bar at 200 rpm. The PBS medium was replaced after 8, 24, 72, 168, and 240 h to reproduce the sink condition [34]. For sample collection, 20-μL aliquots were collected at 0, 2, 4, 6, 8, 24, 48, 72, 168, 240 and 336 h. The samples were diluted 10× with ACN, and the FEN concentrations were calculated by HPLC. All experiments were performed in triplicate.

#### 2.2.6. In Vitro Cytotoxicity Assay

The A549 human non-small cell lung cancer cell line was purchased from the American Type Culture Collection (Manassas, VA, USA). The media for growing cells consists of the following: Roswell Park Memorial Institute medium (RPMI 1640) with 1% (*w*/*v*) streptomycin/penicillin and 10% (*v*/*v*) fetal bovine serum. The cells were grown at 37 °C with a 5% CO_2_ atmosphere conditions. A549 cells were seeded in 96-well plates at a density of 5000 cells per well. After 24 h, the cells were treated with free FEN drug (After dissolving in DMSO, it was diluted 1000 times with RPMI) or FEN-loaded micelles. The initial concentration of treated FEN-loaded soluplus^®^ micelles are 205.5 μM, and the initial concentration of treated free FEN is 33.4 μM. Treatment was done by diluting 10 times from the initial concentration (*n* = 6). After 48 h of incubation, cell viability was assessed by the MTT assay. Microplate reader (Spectra Max ID3, Molecular Devices, San Jose, CA, USA) was used to measure the absorbance at 540 nm 4 h after MTT treatment. Data were handled and curves were fitted with GraphPad Prism v. 5 (GraphPad Software, La Jolla, CA, USA).

#### 2.2.7. Clonogenic Assay

A549 cells were seeded in six-well plates at a density of 300 cells per well. After 24 h, the cells were treated with free FEN drug (After dissolving in DMSO, it was diluted 1000 times with RPMI) or FEN-loaded micelles. The treatments included three increasing concentrations and one control group. After 336 h of incubation, the colonies were stained with crystal violet (0.5% *w*/*v*). After 30 min, the crystal violet was gently washed away with water and the colonies were counted.

#### 2.2.8. FEN-Loaded Micelle Stability Test

Stability tests were conducted at 4, 25 and 37 °C, representing cold chain management, room temperature, and human body temperature, respectively. Briefly, the micelles in water was incubated at 4 °C refrigerator, 25 °C room, and 37 °C water bath. At days 0, 1, 2, 5, 7 and 14, micelles were collected from each sample. Micelle size changes and poly-dispersity indices (PDI) were measured using a DLS device, and the experiment was conducted in triplicate.

#### 2.2.9. Pharmacokinetic Study

All animal experiments conducted in this study were approved by the Institutional Animal Care and Use Committee of Chungbuk National University (No. CBNUR-1407-20; 23 July 23 2020). Male Sprague-Dawley rats (7 weeks old) were purchased from Orient Bio Inc. (Seongnam, Korea) and used in all animal experiments. The rats were maintained in ventilated plastic cages filled with aspen shaving and they were given enough water and food. The animals in each group were cannulated and intravenously injected with FEN (2 mg kg^−1^) in 25% Cremophor EL^®^/EtOH (FEN solution) or FEN-loaded Soluplus^®^ micelles. Blood was then collected at 2, 5, 15, 30, 60, 120, 240 and 480 min after drug administration and the samples were centrifuged at 3000 rpm for 5 min to obtain the plasma [35]. Heparin was used as an anticoagulant, and 400~450 µL of blood was collected through the femoral artery. The samples were immediately frozen at −70 °C and maintained in deep freeze until analysis. A non-compartmental model was used to calculate the relevant pharmacokinetic parameters for FEN including total clearance (CL_t_), AUC, volume of distribution (V_d_), and plasma concentration at time zero (C_0_). The curve fitting for the calculation of pharmacokinetic parameters was carried out using the Sigma Plot V 10.0. (Systat Software, San Jose, CA, USA).

#### 2.2.10. Biological Sample Pretreatment for HPLC Analysis

A 200-µL plasma supernatant sample was extracted with ACN and mixed with 20 µL of IS. The mixture was then centrifuged at 16,600× *g* at 4 °C for 5 min, and 10 µL of supernatant was injected into the HPLC system [35]. Biodistribution was determined by the homogenization method [36]. The sample tissues were homogenized in a glass Potter-Elvehjem-type homogenizer (Ultra Turrax T-25; IKA Works Inc., Staufen, Germany) with a Teflon pestle. The FEN concentration in the supernatant was determined as previously described in Section 2.2.2.

#### 2.2.11. Biodistribution Study

A biodistribution study was performed on the rats after the intravenous injection of FEN solution or FEN-loaded micelles (2 mg kg^−1^). The experiment was conducted twice to observe temporal changes (*n* = 3). The rats were euthanized using CO_2_ gas 1 and 8 h after the first injection, and their kidneys, livers, hearts, spleens, and lungs were excised. The samples were washed with DPBS and stored at −70 °C until the subsequent analysis.

#### 2.2.12. In Vivo Toxicity Assay

Five groups of rats (*n* = 6 per group) were used to assess in vivo toxicity. The rats were intravenously injected with 25% Cremophor EL^®^/EtOH solution containing 2 mg kg^−1^ FEN, 25% polysorbate 80 (Tween 80^®^) solution containing 2 mg kg^−1^ FEN, 25% DMA solution containing 2 mg kg^−1^ FEN, or FEN-loaded Soluplus^®^ micelle solution containing 2 mg kg^−1^ FEN. Change in body weight was measured every 2 days for 14 days. Intravenous injections were performed on days 0, 4, and 8. Toxicity was defined as > 10% loss of total body weight, evidence of discomfort, abnormal behavior, or death. Body weight changes were normalized and displayed as percentages. The initial weight was taken to be 100%. The rats were euthanized in response to serious disability and at the end of the experiment [37].

#### 2.2.13. Statistics

For statistical processing of all data used, Student’s *t*-test or ANOVA of GraphPad Prism v 5.0 was used.

## 3. Results

### 3.1. Physicochemical Characterization of FEN-Loaded Micelles

**Figure 2:**
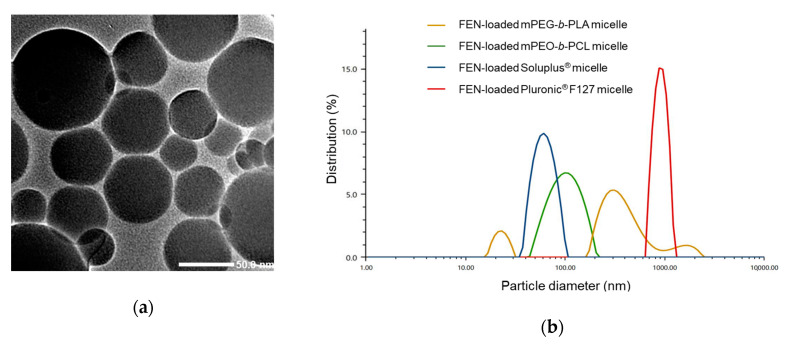
Particle size analysis of the various formulations in this study. (**a**) Transmission electron microscopy (TEM) image of FEN-loaded Soluplus^®^ micelles. (**b**) Size distribution of micelles made of four different polymers.

The DL (%), EE (%), and particle sizes of the FEN-loaded micelles prepared with various polymers (Soluplus^®^, mPEG-*b*-PLA, Pluronic^®^ F127, and mPEO-*b*-PCL) are listed in Table 1. After freeze-drying, the unincorporated drug or polymer was removed by centrifugation and passed through a 0.2-μm filter. As FEN has low water solubility, only small amounts of it would be present in the water. Hence, FEN would be well encapsulated in polymeric micelles [38,39]. DLS analysis revealed that the average particle size of the FEN-loaded Soluplus^®^ micelles was 68.3 nm. In contrast, the other polymeric micelles prepared with mPEG-*b*-PLA, Pluronic^®^ F127, and mPEO-*b*-PCL were much larger (Figure 2). Pluronic^®^ F127 and mPEG-*b*-PLA did not have nano-particle attributes as they were > 300 nm in size. In addition, we confirmed the formation of Soluplus^®^ micelles using TEM. The average particle size in TEM image measured by ImageJ software was 65.6 ± 26.2 nm.

### 3.2. In Vitro Drug Release Profile

**Figure 3:**
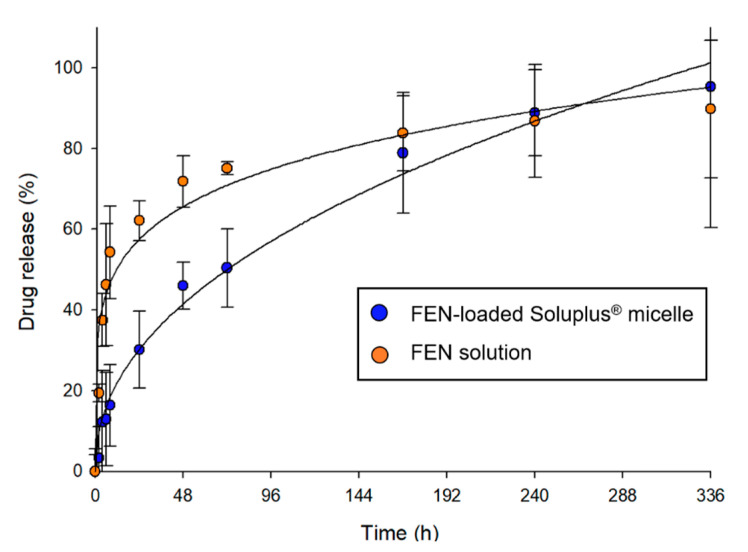
In vitro release profile of fenbendazole (FEN) from Soluplus^®^ micelles and 25% Cremophor EL^®^/EtOH solution at 37 °C.

The in vitro drug release profiles of FEN-loaded Soluplus^®^ micelle and FEN dissolved in 25% Cremophor EL^®^/EtOH solution are shown in Figure 3. After 6 h, the release rates were 12.9% for the micelle formulation and 46.3% for the solution. At ≤ 72 h, the release rates were 50.4% for the micelle and 75.1% for the solution. Over the first 168 h, the solution released FEN significantly faster than the micelle.

### 3.3. In Vitro Cytotoxicity Assay

**Figure 4:**
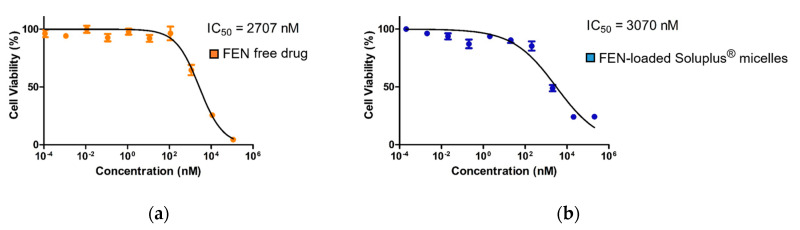
In vitro MTT assay results for the (**a**) free fenbendazole (FEN) drug and (**b**) FEN-loaded Soluplus^®^ micelle treatments of the A549 human non-small cell lung cancer (NSCLC) cell line.

The free FEN drug treatment IC_50_ = 2707 nM, whereas the FEN-loaded Soluplus^®^ micelle treatment IC_50_ = 3070 nM (Figure 4).

### 3.4. Clonogenic Assay

**Figure 5:**
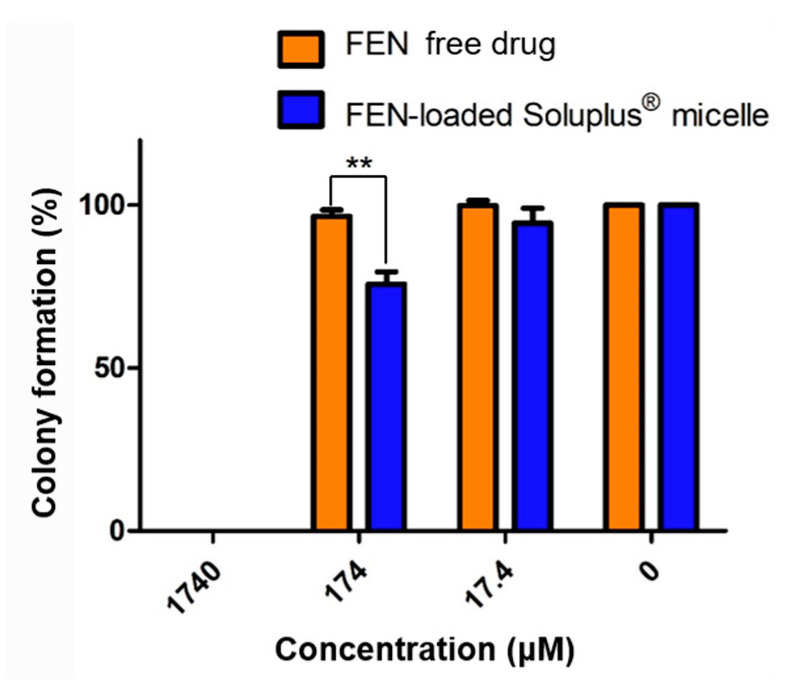
In vitro clonogenic assay of free fenbendazole (FEN)- and FEN-loaded Soluplus^®^ micelle-treated A549 cell line, ** *p* < 0.01.

We performed a clonogenic assay to determine the long-term inhibitory efficacy of the treatments against cell reproduction. The cells formed no colonies at 1740 μM. Colony formation was slightly suppressed in the micelle-treated group at 174 μM. Above 17.4 μM, colony formation was not significantly inhibited (Figure 5).

### 3.5. Stability Test of FEN-Loaded Soluplus^®^ Micelles

**Figure 6:**
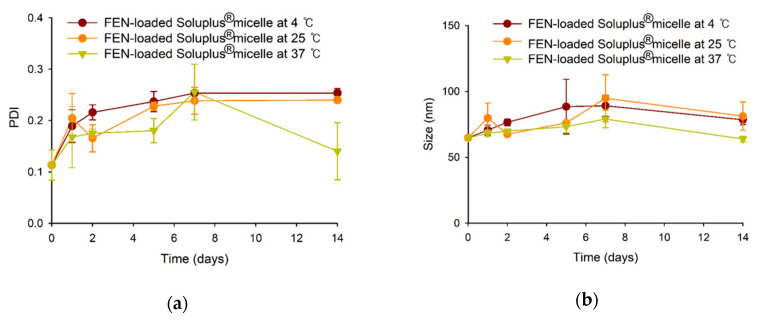
PDI (**a**) and size (**b**) changes of FEN-loaded Soluplus^®^ micelles at various temperature.

FEN-loaded Soluplus^®^ micelles were stable for 2 weeks at 4, 25, and 37 °C. In all cases, the size was ≤100 nm, and the PDI was ≤0.3 (Figure 6).

### 3.6. Pharmacokinetics of FEN Solution and FEN-Loaded Soluplus^®^ Micelles in Rats

**Figure 7:**
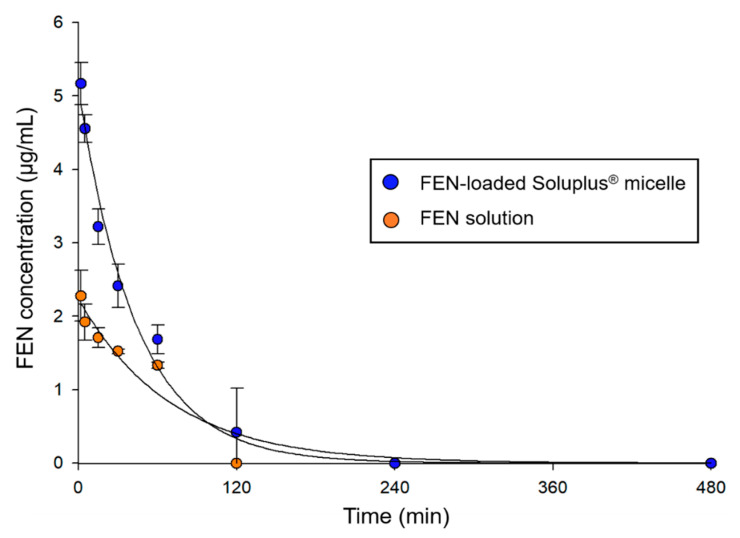
Plasma fenbendazole (FEN) concentration vs. time profile after intravenous injection of FEN-loaded Soluplus^®^ micelles and FEN dissolved in 25% Cremophor EL^®^/EtOH solution.

The plasma concentration–time profiles of the FEN solution and FEN-loaded Soluplus^®^ micelles are shown in Figure 7. The plasma FEN concentration rapidly decreased after the intravenous injection of FEN solution and FEN-loaded Soluplus^®^ micelles. However, FEN-loaded Soluplus^®^ micelles were detected 2 h after injection, whereas the FEN solution was detected within 1 h. At 2 h, no FEN solution was found as its concentration was below the limit of detection (LOD). The pharmacokinetic parameters (Table 2) were calculated assuming a non-compartment model. The AUC and C_0_ were approximately 1.5-fold and more than 2-fold higher for the micelle formulation than for the solution, respectively. Moreover, the CL_t_ and V_d_ were 1.4-fold and more than 2-fold lower for the micelle formulation than for the solution, respectively.

### 3.7. Biodistribution of FEN Solution and FEN-Loaded Soluplus^®^ Micelles in Rats

**Figure 8:**
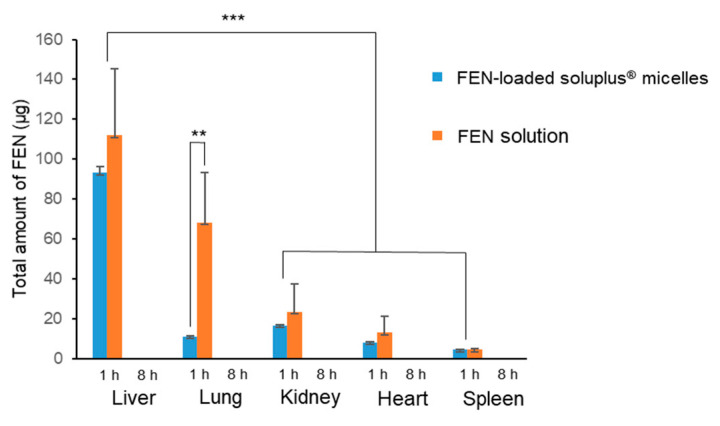
Total amount of fenbendazole (FEN) in each organ at 1 and 8 h after administration of FEN in 25% Cremophor EL^®^/EtOH solution and FEN-loaded Soluplus^®^ micelles, ** *p* < 0.01, *** *p* < 0.001.

Figure 8 shows the total drug distribution in each organ measured 1 and 8 h after the intravenous injection of FEN solution and FEN-loaded Soluplus^®^ micelles. After 1 h, the organ distribution of the drug was lower in micelles than in solution in all other organs except the spleen. After 8 h, both the solution and the micelles were present below the LOD in all organs. In particular, a significantly higher amount of drug was detected in the lungs 1 h after the administration of the solution than 1 h after the administration of the micelles. Additionally, when organs were obtained, some hemolysis was observed in the lungs (data not shown).

### 3.8. In Vivo Toxicity Assay

**Figure 9:**
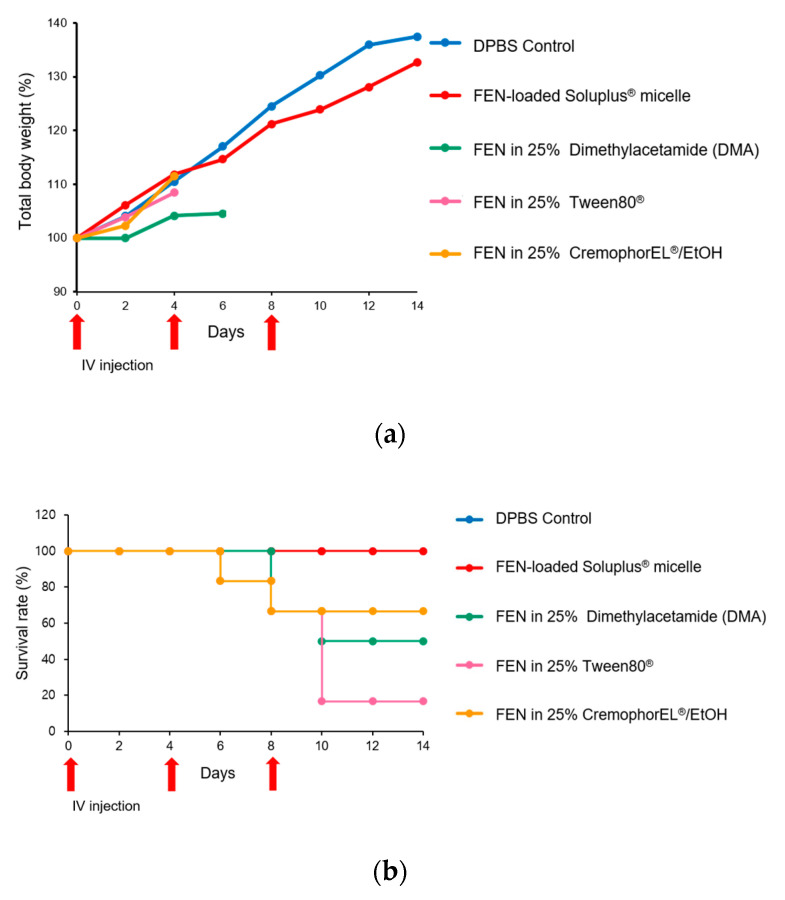
Relative rat body weight changes and survival rates after three intravenous injections of Dulbecco’s phosphate-buffered saline (DPBS) control, fenbendazole (FEN) dissolved in 25% Cremophor EL^®^/EtOH, FEN dissolved in 25% dimethylacetamide (DMA), FEN dissolved in 25% Tween 80^®^, or FEN-loaded Soluplus^®^ micelles on days 0, 4, and 8 (All doses are equal to 2 mg·kg^−1^). (**a**) Relative daily body weight change. Missing data points indicate death of an animal. (**b**) Kaplan–Meier plot illustrating survival rates.

Figure 9 shows the total body weight changes and the survival rate after three injections of DPBS (control), FEN dissolved in 25% Cremophor EL^®^/EtOH, FEN dissolved in 25% DMA, FEN dissolved in 25% Tween 80^®^, or FEN-loaded Soluplus^®^ micelles. Intravenous doses were administered on days 0, 4, and 8 of the experiment, and the survival rates and total body weights were measured. Compared with the control, the FEN-loaded Soluplus^®^ micelle treatment group presented with 100% survival and higher body weight. One rat each in the Tween 80^®^ and Cremophor EL^®^/EtOH groups died on day 6. In the DMA group, three rats died on day 10. After 2 weeks, half the rats in the DMA group, four rats in the Cremophor EL^®^/EtOH group, and one rat in the Tween 80^®^ group survived. All animals in the micelle and control groups survived.

## 4. Discussion

FEN is a drug used as an anthelmintic. However, in recent years, the potential of the drug as an anticancer drug through various mechanisms and its few side effects are being revealed [5,6]. However, the low solubility and bioavailability poses an obstacle to its use [10]. To overcome this deficiency and obtain the advantages of micelles, we formulated, optimized, and tested polymeric FEN micelles. Here, we tested four different polymers and sought the optimal formulation. We compared their physicochemical properties, including particle size, poly-dispersity index, zeta-potential, and encapsulation efficiency. The present study showed that Soluplus^®^ had the highest EE (%) and DL (%), the most appropriate size, and the lowest PDI. Nanoparticles < 200 nm have numerous drug delivery benefits and excellent micelle-forming ability. Small nanoparticles reduce the incidence of nonspecific interactions, including those that occur in the reticuloendothelial system. Secondly, the intravenous injection of micelles obviates the need for kidney excision and mitigates cancer accumulation via the EPR effect [40]. Also, by obtaining TEM data, we clearly showed that the nanoscale micelles we wanted were formed. All four polymers had negative zeta-potentials. Hence, they created electrostatic repulsion and remained safely within the physiological environment [41]. In vitro release profiles disclosed that the micelle formulation had 3-fold and 1.5-fold slower FEN release rates than the solution at 6 and 72 h, respectively. Micelle formulations can solubilize hydrophobic drugs and impede their rapid drug release, possibly because intermolecular interactions occur between the drug and the hydrophobic micelle core. The lipophilic moiety of Soluplus^®^ consists of polyvinyl caprolactam-polyvinyl acetate, which should hydrophobically interact and form hydrogen bonds with FEN. The MTT assay confirmed the short-term (48 h) cytotoxicity of FEN. The difference between the IC_50_ of free FEN and FEN-loaded Soluplus^®^ micelles was ≈10% according to the MTT assay. The IC_50_ was slightly higher for the micelles than for the free drug as the former had a 48-h release rate of only 11.8%. Nevertheless, the micelles and their drug load were internalized, possibly through endocytosis [30,42]. Therefore, despite the low release rate, it is expected that the difference in effect was not large. The IC_50_ for free FEN drug was 3070 nM (3.07 μM). Curcumin has an IC_50_ range of 5.43–108.69 μM, and its anticancer action is downregulation of the BCL-2 family. 17-AAG has an IC_50_ range of 0.1–2.37 μM, and its anticancer mechanism is the suppression of heat shock protein 90. Hence, the IC_50_ of FEN indicates that the drug has sufficient anticancer efficacy [43,44,45]. The clonogenic assay confirmed the long-term inhibition of cell reproduction. It revealed that the FEN micellar formulation entirely inhibited colony formation at 1740 μM but was also effective at only 174 μM. Furthermore, both the size and PDI of the FEN-loaded Soluplus^®^ micelles remained stable for two weeks at three temperatures. Therefore, this product is appropriate for long-term transport and storage in various situations. The FEN-loaded Soluplus^®^ micelles also maintained stability at 37 °C. This is the temperature inside the body, and in terms of temperature, we may expect stability inside the body. The FEN-loaded Soluplus^®^ micelles had superior pharmacokinetic parameters compared to the solution, including higher AUC and C_0_. This is expected to be owing to the decrease in the volume of distribution and total clearance of FEN. Thus, FEN-loaded Soluplus^®^ micelles may exhibit better bioavailability than the free FEN drug solution at equal doses. The biodistribution study indicated that lung damage could be responsible for the observed high pulmonary drug accumulation in the solution group. Cremophor EL^®^ increased the total numbers of cells and macrophages in the lungs, which might suggest inflammation [46]. Therefore, the observed unknown lung injury may have induced localized FEN accumulation. For the in vivo toxicity assay, three commercially applied or extensively tested solubilizing agents were selected and compared against FEN-loaded Soluplus^®^ micelles. However, all the products may induce side effects and could be hepatotoxic and/or neurotoxic. Here, the toxicity of these agents was adjudged by determining the reduction in body weight and survival rate in rats. In the solubilizing agent groups, ≥ 2 rats died. The tween80^®^ group had the largest number of deaths with five. Three were killed in the DMA group and two in the Cremophor EL^®^/EtOH group. It is inferred that this death was caused by the toxicity of solubilizing agent. In contrast, none of the rats in the control and FEN-loaded Soluplus^®^ micelles groups died and they presented with consistent weight gain. Therefore, the FEN-loaded Soluplus^®^ micelles may be considered relatively low toxicity.

## 5. Conclusions

Here, we tested various excipients to solubilize the veterinary anthelmintic FEN. It was recently discovered that this drug also has anticancer efficacy. We selected the Soluplus^®^ micelle by optimization experiments and evaluated its physicochemical properties including particle diameter, zeta-potential, encapsulation efficiency, and drug release. We also conducted pharmacokinetic and biodistribution studies on rats intravenously injected with FEN-loaded Soluplus^®^ micelles. The micellar formulation had superior bioavailability compared to that of free FEN. The biodistribution results indicate that FEN solution is mainly distributed to the lungs and liver compared to other organs. In addition, FEN solution was more specifically distributed to the lungs than micelles. In vivo toxicity tests showed that the FEN-loaded Soluplus^®^ micelle formulation was less toxic than FEN solubilized with other excipients. As FEN has already demonstrated anticancer efficacy, the nanoparticle formulation developed here merits further preclinical research. The ultimate objective is to conduct human clinical safety and efficacy trials on FEN-loaded Soluplus^®^ micelles.

## Figures and Tables

**Table 1:** Characteristics of fenbendazole (FEN)-loaded micelles.

Polymer Amount Used (mg)	FEN Amount Used (mg)	Particle Size (nm)	Poly-Dispersity Index (PDI)	Zeta-Potential (mV)	Encapsulation Efficiency (EE %)	Drug Loading (DL %)
mPEO-*b*-PCL 100 mg	1.0	96.8 ± 3.8	0.14 ± 0.01	−0.4 ± 0.1	24.7 ± 2.0	0.2 ± 0.02
mPEG-*b*-PLA 100 mg	1.0	347.7 ± 40.4	0.26 ± 0.04	−9.5 ± 2.1	56.8 ± 2.8	0.6 ± 0.03
Pluronic F127^®^ 100 mg	1.0	1566.5 ± 157.8	0.23 ± 0.09	−4.6 ± 0.1	N.D.^a^	N.D.^a^
Soluplus^®^ 50 mg	1.0	65.4 ± 2.3	0.11 ± 0.03	−2.4 ± 0.2	58.3 ± 3.1	0.6 ± 0.03
Soluplus^®^ 100 mg	1.0	68.3 ± 0.6	0.01 ± 0.02	−2.3 ± 0.2	85.3 ± 2.9	0.8 ± 0.03

^a^ N.D., not detectable due to precipitation. (*n* = 3; mean ± SD).

**Table 2:** Pharmacokinetic parameters of fenbendazole (FEN) after intravenous injection of FEN-loaded Soluplus^®^ micelles and FEN dissolved in 25% Cremophor EL^®^/EtOH solution.

Parameter	FEN Solution	FEN-Loaded Soluplus^®^ Micelle
AUC ^a^ (min·µg·mL^−^^1^)	156 ± 2.6	234 ± 56.9
C_0_^b^ (µg·mL^−^^1^)	2.2 ± 0.3	5.1 ± 0.2
CL_t_^c^ (mL·kg^−^^1^·min)	12.8 ± 0.2	9.0 ± 1.9
Vd ^d^ (mL·kg^−^^1^)	906 ± 112	393 ± 12.9

^a^ AUC, area under the curve; ^b^ C_0_, plasma concentration at time zero; ^c^ CL_t_, total clearance; ^d^ Vd, volume of distribution.

## Supplementary Materials

The following are available online at https://www.mdpi.com/1999-4923/12/10/1000/s1, Figure S1: Representative chromatograms of fenbendazole (FEN) and genistein (internal standard [IS]) in stock solution and biological plasma sample.
