# Supplementary material for: Physicochemical, Pharmacokinetic, and Toxicity Evaluation of Soluplus® Polymeric Micelles Encapsulating Fenbendazole
Source: Pharmaceutics. 2020 Oct 21;12(10):1000. doi: 10.3390/pharmaceutics12101000 (PMC7589096; doi:10.3390/pharmaceutics12101000)
Supplement: Supplementary file 1 [file pharmaceutics-12-01000-s001.pdf]

pharmaceutics

Supplementary Materials: Physicochemical, Pharmacokinetic, and Toxicity Evaluation of Soluplus® Polymeric Micelles Encapsulating Fenbendazole

Ik Sup Jin, Min Jeong Jo, Chun-Woong Park, Youn Bok Chung, Jin-Seok Kim and Dae Hwan Shin*

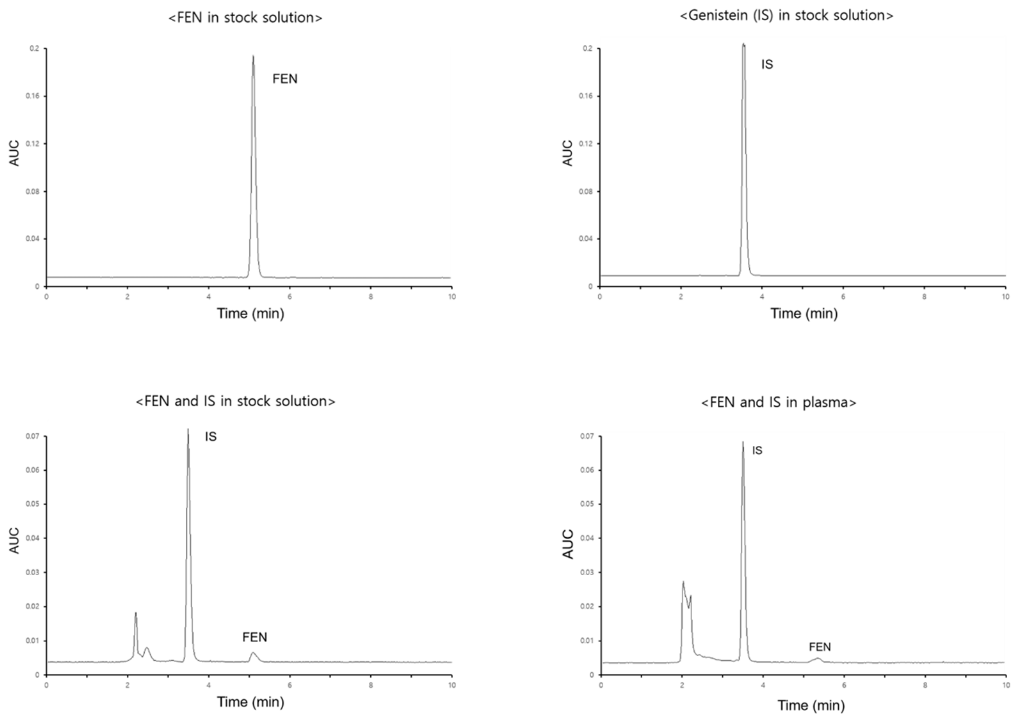

Figure S1. Representative chromatograms of fenbendazole (FEN) and genistein (internal standard [IS]) in stock solution and biological plasma sample.
